# The Novel Immunosuppressive Protein Kinase C Inhibitor Sotrastaurin Has No Pro-Viral Effects on the Replication Cycle of Hepatitis B or C Virus

**DOI:** 10.1371/journal.pone.0024142

**Published:** 2011-09-01

**Authors:** Thomas von Hahn, Andreas Schulze, Ivan Chicano Wust, Benjamin Heidrich, Thomas Becker, Eike Steinmann, Fabian A. Helfritz, Katrin Rohrmann, Stephan Urban, Michael P. Manns, Thomas Pietschmann, Sandra Ciesek

**Affiliations:** 1 Department of Gastroenterology, Hepatology and Endocrinology, Hannover Medical School, Hannover, Germany; 2 Department of Infectious Diseases, Molecular Virology, Otto-Meyerhof-Zentrum, University Hospital Heidelberg, Heidelberg, Germany; 3 Department of Visceral and Transplant Surgery, Hannover Medical School, Hannover, Germany; 4 Division of Experimental Virology, TWINCORE, Centre for Experimental and Clinical Infection Research; a joint venture between the Medical School Hannover (MHH) and the Helmholtz Centre for Infection Research (HZI), Hannover, Germany; Institut Pasteur Korea, Korea

## Abstract

The pan-protein kinase C (PKC) inhibitor sotrastaurin (AEB071) is a novel immunosuppressant currently in phase II trials for immunosuppression after solid organ transplantation. Besides T-cell activation, PKC affects numerous cellular processes that are potentially important for the replication of hepatitis B virus (HBV) and hepatitis C virus (HCV), major blood-borne pathogens prevalent in solid organ transplant recipients. This study uses state of the art virological assays to assess the direct, non-immune mediated effects of sotrastaurin on HBV and HCV. Most importantly, sotrastaurin had no pro-viral effect on either HBV or HCV. In the presence of high concentrations of sotrastaurin, well above those used clinically and close to levels where cytotoxic effects become detectable, there was a reduction of HCV and HBV replication. This reduction is very likely due to cytotoxic and/or anti-proliferative effects rather than direct anti-viral activity of the drug. Replication cycle stages other than genome replication such as viral cell entry and spread of HCV infection directly between adjacent cells was clearly unaffected by sotrastaurin. These data support the evaluation of sotrastaurin in HBV and/or HCV infected transplant recipients.

## Introduction

Worldwide about half a billion people are chronically infected with the hepatitis B virus (HBV), hepatitis C virus (HCV) or both and thus at risk for cirrhosis and hepatocellular carcinoma. HBV and HCV pose a particularly vexing clinical problem in organ transplant recipients [1]. HBV- or HCV-associated end stage liver disease is the leading indication for orthotopic liver transplantation (OLT). In the case of HCV re-infection of the graft is near universal. Recurrent hepatitis C rapidly progresses to cirrhosis and thus frequently necessitates re-transplantation [2]. Of the hemodialysis population 0–10% and 10–65% are infected with HBV or HCV, respectively [3].

Immunosuppression itself results in enhanced viral replication, but as shown by us and others some immunosuppressive agents have additional direct non-immune mediated effects on viral replication: while cyclosporine A (CsA) and its derivatives are potent inhibitors of HCV-replication and are now being developed as anti-viral drugs [4], glucocorticoids have been found to directly stimulate a glucocorticoid-responsive element present in the HBV genome [5] and to enhance HCV-infection of liver cells [6]. To design optimal immunosuppressive regimens in transplant recipients with viral hepatitis it is important to be aware of these properties.

Sotrastaurin (AEB071) is a novel immunosuppressive drug currently in phase I and II clinical trials for post transplant immunosuppression [7] and may also provide a new therapeutic option for psoriasis [8]. It inhibits multiple classical and novel members of the protein kinase C (PKC) family resulting in decreased T-lymphocyte activation [9]. However PKC-family members have a plethora of additional effects on non-immune cells including hepatocytes. The replication of HBV, a DNA virus of the *Hepadnaviridae* family, and HCV, a plus-strand RNA-virus of the *Flaviviridae* family, is intricately linked to PKC-dependent cellular processes [10], [11], [12]. Yet, whether sotrastaurin has a direct effect on HBV- and/or HCV-replication has not been addressed experimentally.

Using cell based *in vitro* infection systems capable of recapitulating the entire replication cycle of these viruses we observed no stimulatory effect of sotrastaurin on HBV or HCV at any drug concentration tested. These data indicate that sotrastaurin may be a viable option to achieve immunosuppression in HBV and/or HCV infected solid organ transplant recipients.

## Materials and Methods

### Drugs

Sotrastaurin (AEB071) and cyclosporin A were provided by Novartis, Basel, Switzerland. Tacrolimus and Bisindolylmaleimide I (BIM-I) were purchased from Sigma-Aldrich (Seelze, Germany). All were dissolved in dimethyl sulfoxide (DMSO).

### Plasmids

The subgenomic JFH1 NS3-5B HCV reporter genome (SG-JFH1-Luc) that encodes firefly luciferase as a sensitive marker of viral genome replication and the full length HCV reporter genome Jc1-Luc also encoding firefly luciferase and a chimeric JFH1 (genotype 2a; GenBank accession no AB047639) and J6/CF (genotype 2a; GenBank accession no AF177036) HCV genome have been described recently [4], [6], [13]. The H77/JFH1 chimeric HCV genome was a kind gift from Jens Bukh (Copenhagen University Hospital, Copenhagen, Denmark) [14].

### Cell Culture

Huh-7.5 [15] and HepaRG [16] cells were cultured as previously described [4], [17]. Four weeks before infection, HepaRG cells were seeded into 12-well plates (Corning, Kaiserslautern, Germany) at 1,25×10^5^ cells per well. Two weeks prior to infection differentiation was induced by adding 0.5% DMSO [17].

### Cell culture grown HCV particle (HCVcc) assays

Assays for HCV genome replication, production of newly formed HCVcc and infection of naive cells were performed using HCV strains Jc1-Luc (containing a luciferase reporter) or chimeric H77/JFH1 genomes and highly HCV-susceptible Huh-7.5 human hepatoma cells as previously described [4], [6]. All luciferase assays were performed in duplicate (replication) or quadruplicates (infection). To measure HCV spread between adjacent cells (cell-to-cell route) we performed a modified version of a recently described agarose overlay assay [18] where “donor” Huh-7.5 cells were infected with HCVcc (H77/JFH1 chimera) at a high multiplicity of infection resulting in >99% HCV-positive cells as assessed by NS5A staining. “Target” Huh-7.5 cells were transduced with a pTRIP-tagRFP-NLS-IPS1 reporter construct recently described by Jones and colleagues consisting of a fusion protein of the red fluorescent protein tagRFP, an SV40 nuclear localization sequence (NLS) and the C-terminal part (amino acids 462–540) of the interferon-β promoter stimulator protein 1 (IPS1) [19]. The C-terminus of IPS-1 contains an NS3/4A protease cleavage site upstream of a mitochondrial membrane anchor. HCV infection then results in relocation of the tagRFP reporter from a punctuate cytosolic pattern of fluorescence (uninfected) to the nucleus (infected). HCV-positive donor and HCV-negative target cells were mixed at a 1:1 ratio and seeded at 4×10^4^ cells per well into 12-well plates. The co-culture was overlaid with 1% Seaplaque low melting temperature agarose to prevent virus spread through the cell-free route. After 96 hours agarose was removed, the cells were co-stained with DAPI and the percentage of infected target cells was determined.

### Lentiviral HCV pseudo-particle (HCVpp) assays

Pseudoparticles were generated as previously described in 293T cells by co-transfection of (1) a lentigenome with a GFP reporter; (2) HIV gag-pol and (3) the HCV glycoproteins E1 and E2 of strain H77 or JFH-1 with Fugene (Roche Diagnostics, Mannheim, Germany) [20]. For pseudovirus transduction filtered supernatants were added to the target cells for 6 hours. Reporter expression was measured after 48 hours.

### Cell viability and proliferation assay

To probe for cytotoxic effects, Huh-7.5 cells were stained with a LIVE/DEAD fixable far red dead cell stain kit from Invitrogen (Darmstadt, Germany) according to the manufactures protocol. The cells were washed twice and immediately analysed on a fluorescence-activated cell sorter (FACSCanto II, Becton-Dickinson, Heidelberg, Germany). Data were analysed using FloJo software (Tree Star, Inc. Oregon Corporation, Ashland, OR). Alternatively, the Cytotoxicity Detection Kit PLUS that measures lactate dehydrogenase (LDH) release from damaged cells was used (Roche Diagnostics, Mannheim, Germany). To measure the percentage of proliferating cells we used the BrdU Labeling kit I (Roche Diagnostics, Mannheim, Germany) according to the manufacturer'’s instructions. Cell viability and proliferation assay were done at the same time as the corresponding replication or infectivity measurements unless stated otherwise.

### HBV assays

HBV virions were generated in HepAD38 cells that contain a Tet-regulated HBV genome [21]. Briefly, supernatant from induced (Tet off) AD38 cells was purified by low speed centrifugation, precipitated overnight at 4°C with 6% polyethylene glycol 8000 (PEG 8000; Sigma), concentrated by centrifugation (8000 × *g*, 60 min, 4°C), resuspended in 1/100 of the initial volume of PBS with 12.5% FCS, and frozen in aliquots at −80°C. For infection, differentiated HepaRG cells (see cell culture section above) were inoculated with 500 µl of medium containing 4% PEG 8000, 2% DMSO and 25 µl of the concentrated virus stock. After overnight incubation the cells were washed three times with PBS and further cultivated for at least nine days. HBsAg and HBeAg in the culture supernatant were determined at day 9 by using Abbott AxSYM assays for HBeAg and HBsAg (Abbott, Wiesbaden, Germany).

## Results

### Effect of sotrastaurin on HCV RNA replication and infectivity

**Figure 1:**
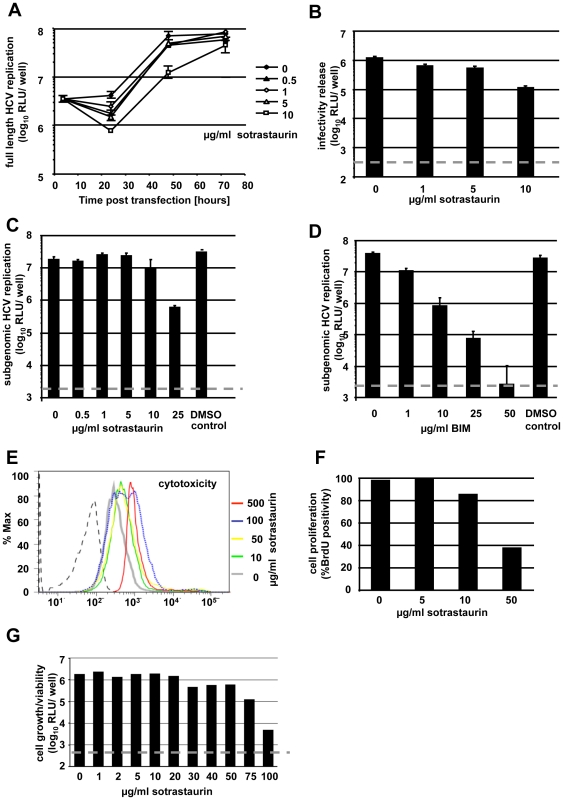
Differential effect of sotrastaurin on HCV RNA replication and virus infection. (A) Replication of HCV in the presence of sotrastaurin at 4, 24, 48 and 72 h post transfection. (B) Inoculation of naïve Huh-7.5 cells using culture fluid from the cells shown in Fig. 1A. After 4 h, cells were supplemented with fresh medium without sotrastaurin. (C+D) Treatment of Huh-7.5 cells harboring a subgenomic HCV replicon (SG-JFH1-Luc) with increasing concentrations of (C) sotrastaurin or (D) BIM-I. The DMSO control corresponds to the DMSO concentration present with the highest drug concentration tested. The luciferase assay was performed 48 hours post transfection. (E) Huh-7.5 cells were treated with increasing amounts of sotrastaurin. After 48 h cell were stained with a live/dead cell stain kit and analyzed by FACS. (F) Percentage of BrdU-positive cells after 48 hours. (G) Luciferase activity as an aggregate measure of viability and proliferation in Huh-7.5 cells stably expressing luciferase under a CMV-promoter treated with sotrastaurin for 72 h.

Huh-7.5 cells were electroporated with the *in vitro* transcribed HCV Jc1-Luc genome and 4 hours later increasing amounts of sotrastaurin were added to the medium. Replication efficiency was assessed 4, 24, 48 and 72 hours after transfection (Fig. 1A). There was no sotrastaurin-induced enhancement of HCV replication detectable. High doses of sotrastaurin (10 µg/ml) decreased HCV RNA replication in transfected cells about 8-fold at 48 hours. At 72 hours replication had reached a plateau for all tested concentrations of sotrastaurin. At this time point RNA levels in cells exposed to 10 µg/ml sotrastaurin reached levels below but close to those measured in untreated cells. In parallel the culture fluid collected 72 hours after transfection was used to inoculate naïve Huh-7.5 cells to probe for an effect on virus production (Fig. 1B). Luciferase activity in the inoculated cells was reduced up to 10-fold. When sotrastaurin was applied to Huh-7.5 cells harboring an NS3-5B subgenomic replicon with a luciferase reporter (SG-JFH1-Luc), again a dose dependent reduction of HCV RNA replication was detected (Fig. 1C). Moreover, the PKC inhibitor BIM-I that has previously been reported to inhibit HCV replication [12], showed the expected mild inhibitory effect on SG-JFH1-Luc in our hands (Fig. 1D).

Given that the sotrastaurin concentrations used in the above experiments are well above the levels achieved in clinical use of the drug, the observed reduction in HCV replication could be due direct anti-viral activity or indirectly mediated through non-specific cytotoxic and/or anti-proliferative effects of the drug. In a standard cytotoxicity assay using a dead cell marker there was no detectable cytotoxicity when cells were exposed to a concentration of 50 µg/ml for 72 hours (Fig. 1E). At 100 and 500 µg/ml about 50% and 100% non-viable cells were detected, respectively. Since sotrastaurin is known to be a potent inhibitor of cell proliferation, we also performed a standard BrdU assay and observed growth arrest in a percentage of cells beginning at concentrations of 10 µg/ml sotrastaurin and above (Fig. 1F). Finally, as an aggregate measure of cell proliferation and viability we used VSV-G pseudoparticles to create a population of Huh-7.5 cells stably expressing firefly luciferase from a CMV-promoter. We treated these cells with different concentrations of sotrastaurin for 72 hours. In line with the above standard assays, a reduction in luciferase activity was detectable at concentrations of 30 µg/ml sotrastaurin and above (Fig. 1G). Taken together, in the Huh-7.5 cells used in our assays sotrastaurin displays anti-proliferative effects at concentrations where a reduction in HCV replication becomes detectable. This suggests that the observed effects on HCV are indirectly mediated through alterations in cellular growth and/or viability rather than being a reflection of a direct anti-viral activity of sotrastaurin.

### Sotrastaurin does not affect HCV entry

**Figure 2:**
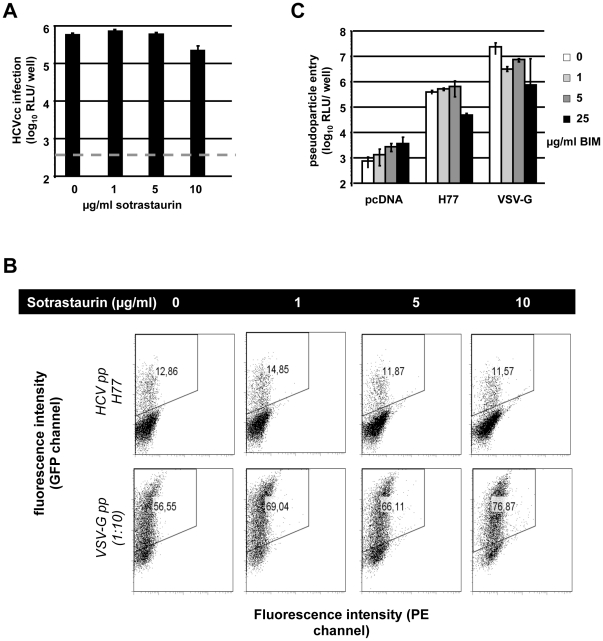
Effect on early HCV replication cycle stages. (A) Infection of Huh-7.5 cells with Jc1-Luc produced in the absence of sotrastaurin. The drug was added for at least 4 h during infection. (B) Strain H77 HCVpp infection of Huh-7.5 cells in the presence or absence of sotrastaurin. Pseudoparticles bearing the glycoprotein of the vesicular stomatitis virus (VSVG) served as controls. (C) Pseudoparticle infection of Huh-7.5 cells in the presence of increasing concentrations of BIM-I. pcDNA represents pseudoparticles devoid of viral glycoproteins.

When using Jc1-Luc viruses prepared in the absence of sotrastaurin to infect naïve Huh-7.5 cells and adding the drug only during inoculation we observed a 3-fold reduction of infectivity (Fig. 2A). Again, this parallels the anti-proliferative effects of high sotrastaurin concentrations described above (Fig. 1F) and is thus not indicative of a specific anti-viral activity. To formally rule out an effect of sotrastaurin on HCV entry, we next used HCV pseudoparticles (HCVpp). HCVpp only test the entry step in the HCV replication cycle without any interference from effects on HCV replication. Here we did not observe an inhibitory effect of sotrastaurin on HCVpp bearing the HCV-glycoproteins E1E2 of genotype 1 or 2 (Fig. 2B and data not shown). Thus even high concentrations of sotrastaurin do not impact on HCV cell entry. The PKC-inhibitor BIM-I, previously described to inhibit HCV RNA replication [12], also had no HCV-specific effect on cell entry (Fig. 2C).

### Sotrastaurin does not affect cell-to-cell transmission of HCV

**Figure 3:**
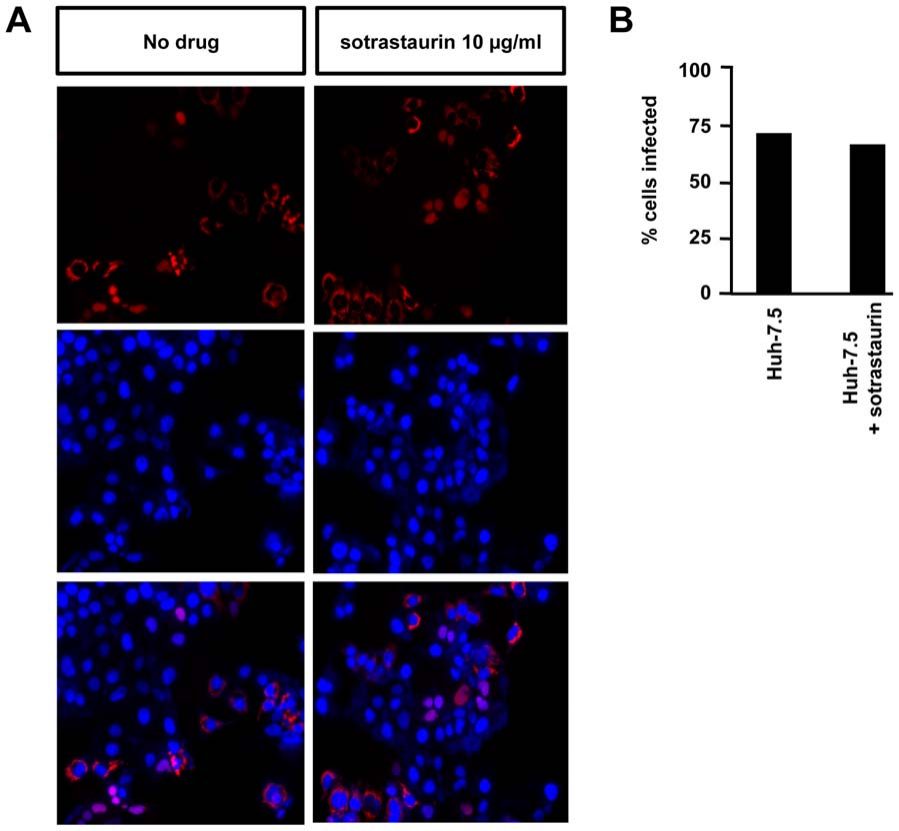
Sotrastaurin does not affect cell-to-cell spread of HCV. (A) HCV-positive donor cells were mixed with HCV-negative target cells under an agarose overlay preventing cell-free virus spread in the presence or absence of sotrastaurin. Target cells contained a tagRFP-reporter that assumes a cytosolic or nuclear localization depending whether the cells are uninfected or infected with HCV. After 96 hours of co-culture nuclei were co-stained with DAPI. (B) Percentage of infected target cells after 96 hours of co-culture.

Besides de novo infection of cells by viral particles in the blood stream HCV is thought to spread within the liver tissue directly to cells adjacent to infected hepatocytes. This cell-to-cell route is thought to circumvent the “extracellular virion” stage of the replication cycle and accordingly be resistant to neutralizing antibodies present in the extracellular space. To test if sotrastaurin has an effect on this route of viral spread we used a co-culture assay (cf. methods section and figure legend) and observed an equal percentage of infected target cells in the presence or absence of 10 µg/ml sotrastaurin (Fig. 3A+B) indicating that cell-to-cell spread is not inhibited.

### Sotrastaurin does not alter the inhibitory effects of CsA on HCV

**Figure 4:**
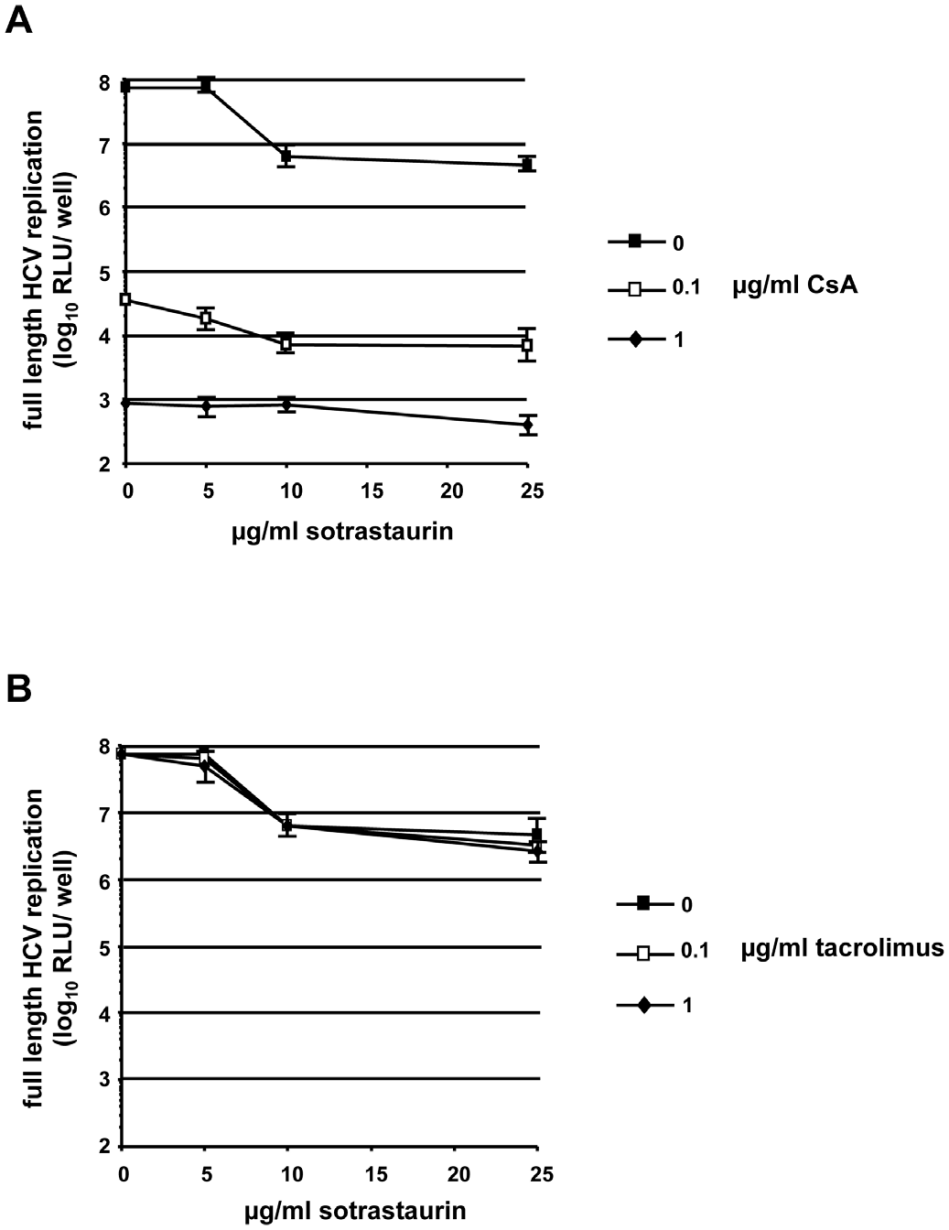
Combination of sotrastaurin with calcineurin inhibitors. (A, B) Effect of different concentrations of sotrastaurin plus (A) CsA or (B) tacrolimus on HCV Jc1-Luc replication at 48 h after electroporation.

Transplant recipients are usually treated with a combination of two or more immunosuppressive drugs. Among potential combination partners of sotrastaurin, CsA is known to have a strong inhibitory effect on HCV RNA replication. To rule out that the combination of CsA with sotrastaurin might influence the antiviral effect of CsA and in order to mimic real-life conditions we tested combinations of sotrastaurin with CsA or tacrolimus, a calcineurin inhibitor with no direct effect on HCV replication. As shown in Figure 4A, CsA inhibited HCV RNA replication irrespective of the presence or absence of sotrastaurin. As expected, tacrolimus had no effect on HCV RNA replication (Fig. 4B). In the presence of either CsA or tacrolimus, there was a reduction in HCV replication when 10 µg/ml or more of sotrastaurin was present which again is likely due to anti-proliferative and/or cytotoxic effects of high drug concentrations.

### Effects of sotrastaurin on the HBV lifecycle

**Figure 5:**
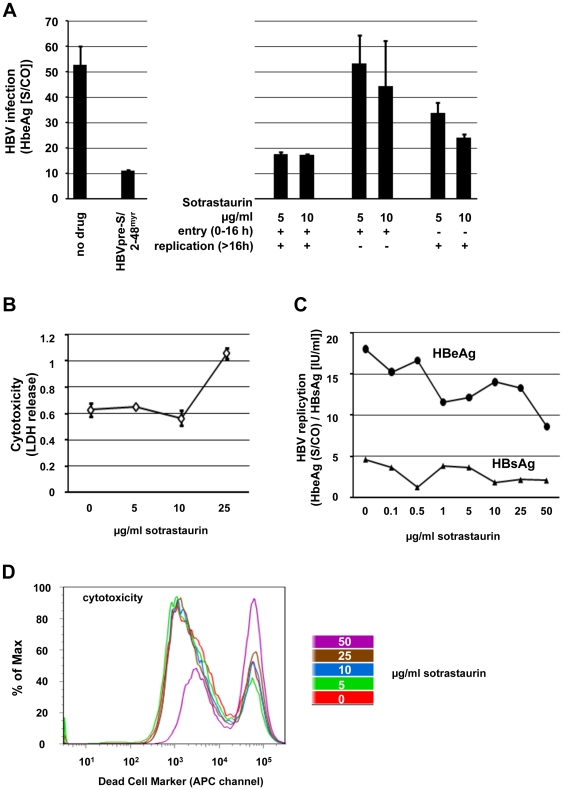
Effects of sotrastaurin on the HBV lifecycle. (A) Differentiated HepaRG cells were infected with HBV virions. Sotrastaurin 5 or 10 µg/ml was present either during inoculation (initial 16 h), during inoculation and infection (throughout), or after inoculation (from 16 h onwards). An inhibitory fragment from the HBV L-protein (HBV pre-S/2-48my) applied during inoculation served as a positive control. All reactions were adjusted to contain the same final concentration of DMSO. HBeAg levels secreted into the cell culture supernatant were determined at day 9 after infection. (B) Cytotoxic effect of sotrastaurin on differentiated HepaRG cells as measured by and LDH release assay. (C) HBV antigens released from AD38 harboring a replicating HBV genome after 72 hours of sotrastaurin treatment. IU – international units; S/CO - signal to cutoff ratio. (D) AD38 cells were treated with increasing amounts of sotrastaurin. After 72 h cells were stained with a live/dead cell stain kit and analyzed by FACS.

To address the question if the novel PKC inhibitor sotrastaurin has an influence on HBV we infected differentiated HepaRGs in the presence or absence of sotrastaurin. HBeAg and HBsAg in the supernatant were measured by ELISA 9 days after inoculation (Fig. 5A and data not shown). To distinguish HBV antigen synthesized in de novo infected cells from antigens in the inoculums, we performed a control experiments where infection was blocked with HBVpre-S/2-48^myr^, a peptide that efficiently blocks HBV entry [17]. As shown in Figure 5A, there was a small (about 3-fold) reduction supernatant antigen levels when sotrastaurin (5 or 10 µg/ml) was present during the entire experiment. In contrast, the presence of sotrastaurin only during the initial 16 hours had no effect, suggesting that HBV entry is not affected by the drug. When sotrastaurin was added after the initial 16 hour inoculation period there was a modest reduction of the infection signal. An enhancement of HBV infection was not observed in any case. In a cytotoxicity assay measuring LDH release no cytotoxic effect of sotrastaurin was detected at concentrations below 25 µg/ml (Fig, 5B). In AD38 cells harboring a replicating HBV genome there was only a minimal effect on HBeAg release detectable while HBsAg release was completely unaffected by sotrastaurin concentrations up to 50 µg/ml (Fig. 5C). In a standard cytotoxicity assay using a dead cell marker there was an increase in the percentage of non-viable cells beginning at 50 µg/ml sotrastaurin exposure for 72 hours. (Fig. 5D).

## Discussion

The pan-PKC inhibitor sotrastaurin (AEB071) is a novel immunosuppressive drug. Currently phase II trials evaluate the combination of sotrastaurin and other immunosuppressive drugs in the post solid organ transplant setting (liver and kidney). Patients with viral hepatitis as yet are excluded from these trials.

To our knowledge this is the first experimental study testing for non-immune mediated effects of sotrastaurin on HBV or HCV replication. The most important finding of this study is that sotrastaurin does not upregulate HBV or HCV replication in any of our assay. These results support that sotrastaurin may be a viable option for immunosuppression in HBV and/or HCV infected organ transplant recipients.

We found that sotrastaurin reduces HCV replication at levels of 10 µg/ml and above which is equivalent to the concentrations where anti-proliferative effects become detectable (10 µg/ml). Overt cytotoxicity was seen at only slightly higher concentration (50-100 µg/ml). Hence we believe that sotrastaurin has no specific activity against HCV, but rather affects the intracellular milieu in a way that indirectly reduces viral replication. It is even conceivable that the presence of the replicating viral genome makes cells more vulnerable to pharmacologically induced apoptosis as has been shown for HCV and actinomycin D and staurosporine [22]. Although we did not evaluate the rate of apoptosis, similar mechanisms, i.e. HCV aggravating anti-proliferative or pro-apoptotic effects of sotrastaurin, could be at play here. Clearly, the sotrastaurin concentrations needed to achieve any reduction in viral replication are much higher than those clinically achieved in humans where the pre-dosing levels of sotrastaurin in kidney transplant recipients are reported in the range of 0.2–1 µg/ml [23]. Thus a virologic, non-immune mediated effect of sotrastaurin on HCV replication in correctly dosed patients is unlikely and – if existent – of no clinical relevance. Murakami and colleagues have reported a mild inhibitory effect on HCV by the PKC-inhibitor BIM-I [12] which we have also observed. BIM-I primarily targets classical PKC isoforms and, to our knowledge, is not under investigation for any clinical application. What part of the HCV replication cycle is affected by BIM-I has not been reported, but our data suggest that RNA replication rather than cell entry is reduced. This apparent difference between the PKC-inhibitors BIM-I and sotrastaurin could well be due to a distinct profile of activity against different PKC isoforms.

In the case of HBV 5 or 10 µg/ml sotrastaurin reduced intracellular genome replication while cytotoxic effects became apparent at 25 µg/ml. Presence of the drug only during cell entry had no effect and its administration only after cell entry has been completed (16 hours) had a very small effect. Given this as well as the negligible effect on HBV in AD38 cells where replication is already well established before the drug is administered, one could speculate that it is the initiation of viral replication that is most sensitive to sotrastaurin. The HBV assays employed do not permit sensitive detection of effects on new particle production since new particle assembly in differentiated HepaRG cells is thought to be very low. The effect of sotrastaurin on HBV appears small and its mechanism is unclear. Although there was no cytotoxicity detectable at the concentrations where the reduction in HBV replication was seen cytotoxicity became apparent at only slightly higher concentrations and it is certainly possible that non-specific perturbations of the intracellular milieu at slightly sub-toxic concentrations adversely affect viral replication. However, the viral transcriptional activators HBx and PreS2-activator large surface protein have both been reported to trigger signaling processes involving PKC so that an anti-viral effect of PKC-inhibition by sotrastaurin would also be plausible [10], [24], [25].

Based on our data we can rule out a direct pro-viral effect of sotrastaurin on either HBV or HCV at any concentration tested. This is important information when considering the use of this immunosuppressant in HBV- and/or HCV-positive recipients of solid organ transplants. Differently from what has been reported for the PKC inhibitor BIM-I we have no evidence for any anti-HCV effect of sotrastaurin even at drug concentrations well above what is clinically achieved in transplant recipients taking sotrastaurin for immunosuppression. Based on our data a small inhibitory effect of sotrastaurin on HBV replication is possible albeit only at concentrations well above what is clinically achieved. Thus based on our findings we do not expect any clinically significant non-immune mediated pro- or anti-viral effect of sotrastaurin on either HBV or HCV.

## Acknowledgments

We are grateful to Takaji Wakita for the JFH1 isolate, to Jens Bukh for the J6CF and the H77/JFH1 genomes, and to Charles Rice for Huh-7.5 cells. We thank Heiner Wedemeyer for critical reading of the manuscript and Novartis (Basel, Switzerland) for providing cyclosporin A and sotrastaurin (AEB071).

## References

[1] Huskey J, Wiseman AC (2011). Chronic viral hepatitis in kidney transplantation.. Nat Rev Nephrol.

[2] Brown RS (2005). Hepatitis C and liver transplantation.. Nature.

[3] Vallet-Pichard A, Fontaine H, Mallet V, Pol S (2011). Viral hepatitis in solid organ transplantation other than liver..

[4] Ciesek S, Steinmann E, Wedemeyer H, Manns MP, Neyts J (2009). Cyclosporine A inhibits hepatitis C virus nonstructural protein 2 through cyclophilin A.. Hepatology.

[5] Tur-Kaspa R, Burk RD, Shaul Y, Shafritz DA (1986). Hepatitis B virus DNA contains a glucocorticoid-responsive element.. Proc Natl Acad Sci U S A.

[6] Ciesek S, Steinmann E, Iken M, Ott M, Helfritz FA (2010). Glucocorticosteroids increase cell entry by hepatitis C virus.. Gastroenterology.

[7] Manicassamy S (2009). Sotrastaurin, a protein kinase C inhibitor for the prevention of transplant rejection and treatment of psoriasis.. Curr Opin Investig Drugs.

[8] Skvara H, Dawid M, Kleyn E, Wolff B, Meingassner JG (2008). The PKC inhibitor AEB071 may be a therapeutic option for psoriasis.. J Clin Invest.

[9] Evenou JP, Wagner J, Zenke G, Brinkmann V, Wagner K (2009). The potent protein kinase C-selective inhibitor AEB071 (sotrastaurin) represents a new class of immunosuppressive agents affecting early T-cell activation.. J Pharmacol Exp Ther.

[10] Kekule AS, Lauer U, Weiss L, Luber B, Hofschneider PH (1993). Hepatitis B virus transactivator HBx uses a tumour promoter signalling pathway.. Nature.

[11] Mee CJ, Farquhar MJ, Harris HJ, Hu K, Ramma W (2010). Hepatitis C virus infection reduces hepatocellular polarity in a vascular endothelial growth factor-dependent manner.. Gastroenterology.

[12] Murakami Y, Noguchi K, Yamagoe S, Suzuki T, Wakita T (2009). Identification of bisindolylmaleimides and indolocarbazoles as inhibitors of HCV replication by tube-capture-RT-PCR.. Antiviral Res.

[13] Steinmann E, Penin F, Kallis S, Patel AH, Bartenschlager R (2007). Hepatitis C virus p7 protein is crucial for assembly and release of infectious virions.. PLoS Pathog.

[14] Gottwein JM, Scheel TK, Jensen TB, Lademann JB, Prentoe JC (2009). Development and characterization of hepatitis C virus genotype 1-7 cell culture systems: role of CD81 and scavenger receptor class B type I and effect of antiviral drugs.. Hepatology.

[15] Blight KJ, McKeating JA, Rice CM (2002). Highly permissive cell lines for subgenomic and genomic hepatitis C virus RNA replication.. J Virol.

[16] Gripon P, Rumin S, Urban S, Le Seyec J, Glaise D (2002). Infection of a human hepatoma cell line by hepatitis B virus.. Proc Natl Acad Sci U S A.

[17] Schulze A, Schieck A, Ni Y, Mier W, Urban S (2010). Fine mapping of pre-S sequence requirements for hepatitis B virus large envelope protein-mediated receptor interaction.. J Virol.

[18] Baldick CJ, Wichroski MJ, Pendri A, Walsh AW, Fang J (2010). A novel small molecule inhibitor of hepatitis C virus entry.. PLoS Pathog.

[19] Jones CT, Catanese MT, Law LM, Khetani SR, Syder AJ (2010). Real-time imaging of hepatitis C virus infection using a fluorescent cell-based reporter system.. Nat Biotechnol.

[20] Evans MJ, von Hahn T, Tscherne DM, Syder AJ, Panis M (2007). Claudin-1 is a hepatitis C virus co-receptor required for a late step in entry.. Nature.

[21] Ladner SK, Otto MJ, Barker CS, Zaifert K, Wang GH (1997). Inducible expression of human hepatitis B virus (HBV) in stably transfected hepatoblastoma cells: a novel system for screening potential inhibitors of HBV replication.. Antimicrob Agents Chemother.

[22] Nomura-Takigawa Y, Nagano-Fujii M, Deng L, Kitazawa S, Ishido S (2006). Non-structural protein 4A of Hepatitis C virus accumulates on mitochondria and renders the cells prone to undergoing mitochondria-mediated apoptosis.. J Gen Virol.

[23] Kovarik JM, Steiger JU, Grinyo JM, Rostaing L, Arns W (2011). Pharmacokinetics of sotrastaurin combined with tacrolimus or mycophenolic acid in de novo kidney transplant recipients.. Transplantation.

[24] Kang-Park S, Lee JH, Shin JH, Lee YI (2001). Activation of the IGF-II gene by HBV-X protein requires PKC and p44/p42 map kinase signalings.. Biochem Biophys Res Commun.

[25] Stockl L, Berting A, Malkowski B, Foerste R, Hofschneider PH (2003). Integrity of c-Raf-1/MEK signal transduction cascade is essential for hepatitis B virus gene expression.. Oncogene.

